# Mutant NPM1-Regulated FTO-Mediated m^6^A Demethylation Promotes Leukemic Cell Survival *via* PDGFRB/ERK Signaling Axis

**DOI:** 10.3389/fonc.2022.817584

**Published:** 2022-02-08

**Authors:** Qiaoling Xiao, Li Lei, Jun Ren, Meixi Peng, Yipei Jing, Xueke Jiang, Junpeng Huang, Yonghong Tao, Can Lin, Jing Yang, Minghui Sun, Lisha Tang, Xingyu Wei, Zailin Yang, Ling Zhang

**Affiliations:** ^1^Key Laboratory of Laboratory Medical Diagnostics Designated by the Ministry of Education, School of Laboratory Medicine, Chongqing Medical University, Chongqing, China; ^2^Hematology Oncology Center, Chongqing University Cancer Hospital, Chongqing, China

**Keywords:** acute myeloid leukemia, nucleophosmin 1, *N^6^*-methyladenosine, FTO, PDGFRB, ERK cascade

## Abstract

Acute myeloid leukemia (AML) with nucleophosmin 1 (NPM1) mutations exhibits distinct biological and clinical features, accounting for approximately one-third of AML. Recently, the *N*^6^-methyladenosine (m^6^A) RNA modification has emerged as a new epigenetic modification to contribute to tumorigenesis and development. However, there is limited knowledge on the role of m^6^A modifications in NPM1-mutated AML. In this study, the decreased m^6^A level was first detected and high expression of fat mass and obesity-associated protein (FTO) was responsible for the m^6^A suppression in NPM1-mutated AML. FTO upregulation was partially induced by NPM1 mutation type A (NPM1-mA) through impeding the proteasome pathway. Importantly, FTO promoted leukemic cell survival by facilitating cell cycle and inhibiting cell apoptosis. Mechanistic investigations demonstrated that FTO depended on its m^6^A RNA demethylase activity to activate PDGFRB/ERK signaling axis. Our findings indicate that FTO-mediated m^6^A demethylation plays an oncogenic role in NPM1-mutated AML and provide a new layer of epigenetic insight for future treatments of this distinctly leukemic entity.

## Introduction

Acute myeloid leukemia (AML), a heterogeneous hematologic malignancy, is the most common form of acute leukemia among adults (1). Nucleophosmin 1 (NPM1) mutations represent the most common genetic lesions in AML (2). Among over 50 types of these mutations discovered in exon 12 of the *NPM1* gene, NPM1 mutation type A (NPM1-mA) is identified as the most frequent mutation (3, 4). Considering unique biological and clinical features, AML with NPM1 mutations has been categorized as a distinct entity in the 2016 updated World Health Organization (WHO) classification of AML (5). Despite intense research efforts (6, 7), treatments against this leukemic subtype have not significantly improved (8). Hence, deeper explorations of underlying molecular mechanisms contributing to NPM1-mutated AML and novel therapeutic targets are imperative.

To date, with the development of high-throughput sequencing technology, RNA modifications have been the focus of accumulating studies (9–11). *N*^6^-methyladenosine (m^6^A), methylated at the *N^6^* position of adenosine, has been recognized as the most prevalent internal modification in eukaryotic messenger RNAs (mRNAs), which participates in the regulation of RNA transcription (12), processing (13), translation (14), and degradation (15). It is well accepted that the deposition of m^6^A modifications is dynamic and reversible process. A multicomponent m^6^A methyltransferase complex (MTC) including a core component formed by methyltransferase-like 3 (METTL3)/methyltransferase-like 14 (METTL14) heterodimer (16) and other regulatory factors (WTAP, RBM15/15B, VIRMA, and ZC3H13) (17–19) is responsible for installing these modifications in RNAs. Meanwhile, m^6^A modifications can be removed by α-ketoglutarate-dependent and Fe(II)-dependent demethylases, specifically fat mass and obesity-associated protein (FTO) (20) and alkB homolog 5 (ALKBH5) (21). Additionally, the functions of m^6^A modifications on RNA metabolism depend on specific recognition by m^6^A binding proteins (YTHDF1/2/3, YTHDC1/2, IGF2BP1/2/3, and hnRNPC) (22–24). A rapidly accumulating wealth of studies are delineating the aberrant m^6^A abundance driven by the dysregulation of m^6^A modification enzymes in diverse cancers. Wang et al. (25) reported that inhibition of the m^6^A methylation by depletion of METTL3 and METTL14, enhanced response to anti-programmed cell death-1 (PD-1) treatments in colorectal cancer and melanoma. In another study, FTO downregulation led to an increase of the m^6^A abundance, and overexpression of FTO suppressed the self-renewal of ovarian cancer stem cells (26). Furthermore, depletion of ALKBH5 inhibited the proliferation and invasion capabilities of hepatocellular carcinoma cells and was an independent prognostic factor of worse survival (27). Recently, emerging evidence has revealed that m^6^A regulators are implicated in normal and malignant hematopoiesis. Loss of METTL14 promoted myeloid differentiation of hematopoietic stem/progenitor cells (HSPCs) and AML cells (28). WTAP was reported as an important protein in abnormal proliferation and arrested differentiation of leukemic cells and high WTAP expression predicted poor prognosis in AML (29, 30). RBM15 was essential for the ability of hematopoietic stem cells (HSCs) to contribute normally to adult hematopoiesis (31). Recent studies also revealed that ALKBH5 is required for the occurrence and development of AML and self-renewal of leukemia stem/initiating cells (LSCs/LICs) but dispensable for normal hematopoiesis (32, 33). In addition, it becomes clear that m^6^A binding proteins are crucial to HSC function and AML. YTHDF2 depletion compromised LSCs activity while promoting normal HSC expansion (34). Sheng et al. (35) demonstrated that overexpression of YTHDC1 played an essential role in proliferation and survival of AML both *in vitro* and *in vivo*. Genetic inhibition of IGF2BP1 decreased tumorigenic potential of leukemia cells and led to myeloid differentiation (36). However, the contributions of m^6^A modifications and m^6^A regulators to NPM1-mutated AML development remain unknown. Therefore, it is essential to investigate the biological significance of m^6^A modifications for NPM1-mutated AML.

In the present study, the downregulation of the global m^6^A abundance was first detected and that reduction was triggered by high expression of FTO in NPM1-mutated AML. FTO expression was upregulated in part by NPM1-mA through inhibiting the proteasome pathway. More importantly, FTO facilitated leukemic cell proliferation. Mechanistic investigations demonstrated that FTO activated PDGFRB/ERK signaling axis by its m^6^A RNA demethylase activity. Collectively, our study reveals the oncogenic role of FTO-mediated m^6^A demethylation and provides a new layer of epigenetic insight for future treatments of this distinctly leukemic entity.

## Materials and Methods

### Clinical Samples

The peripheral blood and bone marrow samples of patients with newly diagnosed AML and the healthy donor were obtained from the First Affiliated Hospital of Chongqing Medical University, the Third Affiliated Hospital of Chongqing Medical University, and Chongqing University Cancer Hospital. The experiments were approved by the Ethics Committee of Chongqing Medical University and Chongqing University. This study was performed in compliance with the Declaration of Helsinki. Written informed consents were signed by all subjects for study purposes. The mononuclear cells were enriched by Ficoll mononuclear cell separation solution (Hao Yang Biological Manufacture Co., Ltd, Tianjin, China, #TBD2013CHU05). Briefly, the sample was added to the surface of Ficoll and then centrifuged at 450 g for 30 min to form discrete layers. The mononuclear layer was collected and washed twice with phosphate-buffered saline (PBS). Finally, total RNAs from the mononuclear cells were isolated for the analysis of the global m^6^A abundance and PDGFRB mRNA expression. Details of the clinical characteristics of patients are provided in **Table 1**.

**Table 1:** Clinical characteristics of newly diagnosed AML patients.

Characteristics	Median (range)	No. of cases
Sex		
Female		20
Male		17
Total		37
Median age (years)	49.5 (15-82)	
Younger than 40 y		11
40-60 y		14
Older than 60 y		12
Median WBC, 10^9^/L	61.8 (0.22-347.0)	
Median platelets, 10^9^/L	58.2 (2.0 - 300.0)	
Healthy donor		1
AML		36
AML FAB subtype		
AML without maturation: M1		2
AML with maturation: M2		5
Acute promyelocytic leukemia: M3		2
Acute myelomonocytic leukemia: M4		7
Acute monoblastic or monocytic leukemia: M5		18
Other subtypes		2
Karyotype		
Normal		20
t(8;21)		3
t(15;17)		5
inv(16)		2
Unknown		6
Gene mutations		
*NPM1*		15
*FLT3-ITD*		8
IDH1/IDH2		6
*DNMT3A*		7
*WT1*		12

AML, acute myeloid leukemia; y, year old; WBC, white blood cell; FAB classification, French-American-British classification, a classification of acute leukemia produced by three-nation joint collaboration.

### *N*^6^-Methyladenosine (m^6^A) Dot Blot Assays

Total RNAs from the harvested cells were extracted using the TRIzol reagent (Thermo Fisher Scientific, Waltham, MA, USA, #15596026) following the manufacturer’s protocols. m^6^A dot blot assays were performed essentially as previously reported (37). The extracted RNA samples were diluted to 200 ng/μL, 100 ng/μL, and 50 ng/μL using RNase-free water, followed by denaturation at 95°C for 3 min and chilling on ice immediately. 2 μL denatured RNA samples were spotted and then UV cross-linked to the Hybond-N+ membrane (GE Healthcare, Chicago, IL, USA, #RPN203B). After being blocked with 5% non-fat milk in PBS containing 0.1% Tween-20 (PBST) at room temperature for 2 h, the membrane was incubated with a specific m^6^A antibody (1:1000, Abcam, Cambridge, United Kingdom, #ab284130) at 4°C overnight. Then, the membrane was washed with PBST three times and incubated with horseradish peroxidase (HRP)-conjugated goat anti-rabbit IgG (1:5000, Biosharp, Beijing, China, #BL003A) at room temperature for 1 h. Whereafter, signals were detected using the enhanced chemiluminescence (ECL) solution (Bio-Rad, Hercules, CA, USA, #1705062) and quantified using Image J software (Version 1.8.0). Finally, the membrane stained with methylene blue solution diluted to 0.02% (Solarbio, Beijing, China, #G1301) at room temperature for 15 min, was utilized to ensure equal RNA loading among different groups.

### Date Analysis of the Cancer Genome Atlas (TCGA), Gene Expression Omnibus (GEO), and Beat AML Databases

Gene expression profiles and clinical characteristics of acute myeloid leukemia (AML) patients with normal karyotype were downloaded from the Cancer Genome Atlas (TCGA) database (n=65) and the NCBI Gene Expression Omnibus (GEO) database under the accession numbers GSE14468 (n=187) and GSE15434 (n=251). To filter out m^6^A-related genes from the gene expression matrix, we used R software (Version 3.6.3) and R package biomaRt to annotate the obtained data. The R packages ggplot2 and pheatmap were used to generate m^6^A-related gene expression heatmaps. For Kaplan-Meier survival analysis, all the NPM1-mutated AML patients from TCGA database were divided into high and low gene expression groups with the median value of *FTO*, *ALKBH5*, or *METTL3* level at diagnosis as the cut-off criterion. Overall survival was defined as the time from the date of diagnosis to death due to any cause. The Log-rank test was used to test the difference of overall survival between two groups. For univariate and multivariate analyses, the association between clinicopathological factors (including *FTO* expression) and overall survival of NPM1-mutated AML patients from Beat AML dataset (n=42) was evaluated by Cox proportional hazards regression models. R package survival was used to conduct univariate and multivariate analyses. R package forestplot was used to generate forest plots. For Gene Set Enrichment Analysis (GSEA), all the NPM1-mutated AML patients from GEO dataset GSE14468 were divided into high and low *FTO* expression groups with the median value of *FTO* level as the cut-off criterion. R package clusterProfiler was used to identify the potential signaling pathways related to *FTO* expression in NPM1-mutated AML. All gene sets were selected from the Molecular Signatures Database (MSigDB) and were converted to Gene Matrix Transposed (GMT) format for GSEA analysis and visualization.

### Cell Culture

Human acute myeloid leukemia cell lines OCI-AML3 (carrying NPM1 mutation type A, NPM1-mA) and OCI-AML2 were purchased from Deutsche Sammlung von Mikroorganismen und Zellkulturen GmbH (DSMZ, Braunschweig, NI, Germany) and cultured in RPMI-1640 medium (Thermo Fisher Scientific, #11875093) supplemented with 10% fetal bovine serum (FBS, Thermo Fisher Scientific, #10099141C). Human acute myeloid leukemia cell lines THP-1 and NB4 were purchased from American Type Culture Collection (ATCC, Manassas, VA, USA) and maintained in RPMI-1640 medium containing 10% FBS (Thermo Fisher Scientific, #10091155). To prevent potential contamination, all the mediums were supplemented with 1% Penicillin-Streptomycin solution (Beyotime, Shanghai, China, #C0222). All cell lines were incubated at 37°C in the presence of 5% CO_2_.

### Establishment of Stable Expression Cell Lines

For knockdown analysis, the lentiviral vectors expressing the short hairpin RNA (shRNA) construct targeting NPM1 and shRNA constructs targeting FTO were ordered from GenePharma and Genechem (Shanghai, China), respectively. For overexpression analysis, the lentiviral vectors expressing wild-type NPM1 (NPM1-wt) and NPM1-mA sequence were purchased from Genechem. 1 × 10^5^ leukemic cells per well were plated into a 24-well plate and infected with the above lentiviruses for 72 h with HitransG P (Genechem, #REVG005), followed by puromycin selection (2 μg/mL, Beyotime, #ST551) for 7 d. The puromycin-resistant cells were isolated and propagated for further analyses. The shRNA construct targeting sequence for human NPM1 was 5’-GCCGACAAAGATTATCACTTT-3’ (termed as shNPM1); The shRNA constructs targeting sequences for human FTO were 5’-GACAAAGCCTAACCTACTT-3’ (termed as shFTO #1) and 5’-GAGCTTTGAGTCCTATGCT-3’ (termed as shFTO #2).

### Cell Transfection

The pcDNA3.1 vectors expressing wild-type FTO (FTO-WT), enzymatic activity dead mutant FTO with H231A and D233A two point-mutations (FTO-MUT), and empty vector (Vector) were constructed as previously reported (20) by Genecreate (Wuhan, China). The short interfering RNA (siRNA) targeting PDGFRB (siPDGFRB) and control siRNA were synthesized by Ribobio (Guangzhou, China). For transfections, 2.5 × 10^5^ cells per well were plated into a 24-well plate using Lipofectamine 2000™ Transfection Reagent (Invitrogen, Carlsbad, CA, USA, #11668500) following the manufacturer’s protocols. The siRNA targeting sequence for human PDGFRB was 5’- CAACGAGTCTCCAGTGCTA-3’.

### Reagent Treatments

The inhibitors and activator used in this study are as follows: 100 μg/mL protein synthesis inhibitor cycloheximide (CHX) (Millipore, Burlington, MA, USA, #5.08739) for 0, 6, and 12 h; 20 μM proteasome inhibitor MG132 (Topscience, Shanghai, China, #T2154), 20 μM lysosome inhibitor chloroquine (CQ) (Topscience, #T8689), and 20 μM autophagy activator rapamycin (Selleck, Houston, TX, USA, #S1039) for 8 h; FTO demethylase activity inhibitor meclofenamic acid (MA) (Selleck, #S4295) at 0, 10, 25, 50, and 100 μM for the indicated time points; 5 μM ERK cascade inhibitor U0126 (Topscience, #T6223), 5 μM JNK cascade inhibitor SP600125 (Selleck, #S1460), and 5 μM p38 cascade inhibitor SB203580 (Selleck, #S1076) for 24 h; DMSO was used as a control.

### Quantitative Real-Time PCR (qRT-PCR) Assays

Total RNAs from the harvested cells were isolated with the TRIzol reagent according to the manufacturer’s guidelines. For cDNA synthesis, 1,000 ng of the total RNAs were used in 20 μL reaction volume using PrimeScript™ RT Master Mix (Perfect Real Time) (Takara, Kyoto, Japan, #RR036A). The qRT-PCR analysis was carried out using TB Green™ Premix Ex Taq™ II (Tli RNaseH Plus) (Takara, #RR820A) on a CFX Connect™ real-time system (Bio-Rad). Cycling conditions were 30 s at 95°C for the initial denaturation, and the amplification was performed with 39 cycles of 5 s at 95°C, 30 s at 58°C, and 20 s at 72°C, and finally 10 min at 72°C for the extension. β-actin was used as an internal standard control. Each reaction was run in triplicates. The relative expression level of FTO, PDGFRB, IGF1R, VEGFR, and EGFR mRNA in AML cell lines was analyzed following the 2^-ΔΔCt^ method. Additionally, the relative expression level of PDGFRB mRNA in primary AML blasts were calculated using the 2^-ΔCt^ method. All the primer sequences used are listed in **Table 2**.

**Table 2:** The PCR primer sequences for each gene used in this study.

Genes	Sequences (5’ - 3’)
*FTO*	F: 5’-ACTTGGCTCCCTTATCTGACC-3’
	R: 5’-TGTGCAGTGTGAGAAAGGCTT-3’
*β-actin*	F: 5’-TAGTTGCGTTACACCCTTTCTTG-3’
	R: 5’-TGCTGTCACCTTCACCGTTC-3’
*NPM1-mA*	F: 5’-TGGAGGTGGTAGCAAGGTTC-3’
	R: 5’- CTTCCTCCACTGCCAGACAGA-3’
*NPM1-wt*	F: 5’-ACGGTCAGTTTAGGGGCTG-3’
	R: 5’-CTGTGGAACCTTGCTACCACC-3’
*PDGFRB*	F: 5’-TGATGCCGAGGAACTATTCATCT-3’
	R: 5’-TTTCTTCTCGTGCAGTGTCAC-3’
*IGF1R*	F: 5’-AAAAACCTTCGCCTCATCC-3’
	R: 5’-TGGTTGTCGAGGACGTAGAA-3’
*VEGFR*	F: 5’-GTGATCGGAAATGACACTGGAG-3’
	R: 5’-CATGTTGGTCACTAACAGAAGCA-3’
*EGFR*	F: 5’-AGGCACGAGTAACAAGCTCAC-3’
	R: 5’-ATGAGGACATAACCAGCCACC-3’

F stands for forward; R stands for reverse.

### Western Blot Assays

The treated cells were washed twice with precooled PBS and lysed in the RIPA buffer (Beyotime, #P0013C) containing the protease inhibitor (Bimake, Houston, TX, USA, #B14001) on ice for 30 min, followed by centrifugation at 13,300 rpm at 4°C for 30 min. Then, the supernatant was quantified by the Enhanced BCA Protein Assay Kit (Beyotime, #P0010S) and boiled in 5 × sodium dodecyl sulfate-polyacrylamide gel electrophoresis (SDS-PAGE) loading buffer (Beyotime, #P0015). Subsequently, 50 μg total proteins from each sample were separated on a 12% SDS-PAGE and transferred onto polyvinylidene fluoride (PVDF) membranes (Bio-Rad, #1620177). The membranes were blocked with 5% non-fat milk in tris-buffered saline (TBS) containing 0.02% Tween-20 (TBST) at room temperature for 2 h and incubated with primary antibodies at 4°C overnight. After being washed with TBST three times, the membranes were incubated with secondary antibodies at room temperature for 1 h. Next, signals were detected using the ECL solution and quantified using Image J software. β-actin was used as the internal standard control. Antibodies used for western blotting are as follows: anti-NPM1-mA (1:1000, #PA1-46356) was purchased from Thermo Fisher Scientific; anti-NPM1 (1:1000, #ab52644) was purchased from Abcam; anti-FTO (1:1000, #A5594), anti-ERK (1:1000, #A5029), anti-p-ERK (1:1000, #A5036), anti-JNK (1:1000, #A5005), anti-cleaved Caspase 9 (1:1000, #A5074), anti-Cyclin A2 (1:1000, #A5666), and anti-PDGFRB (1:1000, #A5541) were purchased from Bimake; anti-total Caspase 9 (1:1000, #9504), and anti-p-JNK (1:1000, #4668) were purchased from Cell Signaling Technology (Danvers, MA, USA); anti-β-actin (1:1000, #TA-09) was purchased from ZSGB-BIO (Beijing, China); HRP-conjugated goat anti-rabbit IgG (1:5000, #BL003A) and HRP-conjugated goat anti-mouse IgG (1:5000, #BL001A) were purchased from Biosharp (Beijing, China).

### Ubiquitination Assays

The cells were incubated with 20 μM MG132 for 8 h to induce the ubiquitination of FTO before harvest. After being spiked with the cell lysis buffer (Beyotime, #P0013) containing the protease inhibitor (Bimake, #B14001), the harvested cells were sonicated and rested on ice for 20 min, followed by centrifugation at 13,300 rpm at 4°C for 30 min to remove the cell debris. The total cell lysate was quantified by the Enhanced BCA Protein Assay Kit to normalize the total amounts of the inputs. Afterward, the supernatant was incubated with protein A/G beads (Bimake, #B23201) which were coated with an anti-FTO antibody (1:50, Cell Signaling Technology, #31687) at 4°C overnight. The protein A/G beads combined with immunocomplexes were washed with PBST three times before being boiled in 2 × SDS-PAGE Sample Loading Buffer (Beyotime, #P0015B). The boiled samples were then immunoblotted by an anti-ubiquitin antibody (1:1000, Santa Cruz Biotechnology, Dallas, TX, USA, #sc-8017).

### Cell Counting Kit-8 (CCK-8) Assays

The cell proliferation was assessed with CCK-8 assays. The lentiviral infection cells or the cells transfected with plasmids for 24 h were plated into 96-well plates at a density of 1.5 × 10^4^ cells per well, and subsequently treated with indicated reagents. Then, the cells were cultivated in 100 μL RPMI-1640 medium containing 10% FBS. At the corresponding time point after seeding, each well was spiked with 10 μL CCK-8 solution (Solarbio, #CA1210), and the plate was incubated at 37°C for 2-4 h in the dark. The absorbance value at 450 nm was measured using the microplate reader (BioTeck, CA, USA).

### 5-Ethynyl-2’-Deoxyuridine (EdU) Assays

The cell viability was determined by a BeyoClick™ EdU Cell Proliferation Kit with Alexa Fluor 594 (Beyotime, #C0078S), which measured the rate of DNA replication. In brief, the cells after pretreatment were seeded into a 6-well plate at a density of 1 × 10^6^ cells per well and then exposed to 10 μM EdU solution for 3 h at 37°C before harvest. After being washed twice with cold PBS, the collected cells were prepared into the suspension and evenly coated on the slides, followed by fixation in 4% paraformaldehyde and permeabilization with 0.1% Triton X-100 at room temperature for 15 min in sequence. Subsequently, the slides were washed with PBS three times and covered by enough click reaction solution according to the instructions for 30 min at room temperature in the dark. After being washing three times with PBS, the slides were stained with the DAPI solution for 30 min to label the cell nuclei. Images were captured with a fluorescence microscope (Nikon, Tokyo, Japan) at 200 × magnification. The ratio of EdU-stained cells (with red fluorescence) to DAPI-stained cells (with blue fluorescence) in three randomly selected fields was used to calculate the percentage of EdU positive cells.

### Cell Cycle Assays

The cell cycle was analyzed by Propidium (PI) DNA staining. 1 × 10^6^ cells were washed twice with precooled PBS and fixed in 500 μL precooled 75% ethanol at 4°C overnight, followed by centrifugation. The cell pellet was obtained and added with 100 μL RNase A in the dark and incubation at 37°C for 30 min. Then, the cells were spiked with 400 μL PI (Millipore, #537060) and incubated at 4°C for 30 min away from light. The cell cycle distribution of 1 × 10^4^ cells was assessed using FACSCalibur™ Flow cytometry (BD Biosciences, Piscataway, NJ, USA) and ModFit LT software (Version 5.0).

### Cell Apoptosis Assays

The cell apoptosis was determined by an Annexin V-APC/DAPI Apoptosis Detection Kit (Elabscience, Wuhan, China, #E-CK-A258). 1 × 10^6^ cells were washed twice with precooled PBS, followed by centrifugation. The cell pellet was resuspended in 500 μL diluted Annexin V Binding Buffer, followed by addition of 5 μL Annexin V-APC and 5 μL DAPI staining solution. Then, the cells were incubated at room temperature for 20 min in the dark. FACSCalibur™ Flow cytometry and CytExpert software were used to analyze 1 × 10^4^ stained cells.

### Statistical Analysis

Data from at least three independent experiments were presented as the mean ± standard deviation (SD). Statistical analysis was performed using GraphPad Prism (Version 7.00). Differences between two groups were evaluated using the unpaired Student’s t-test. Differences among multiple groups were compared using the one-way analysis of variance (ANOVA). The Kaplan-Meier estimation and the log-rank test were used to compare the survival difference. *P* < 0.05 was considered statistically significant (*P < 0.05, **P < 0.01, ***P ***<*** 0.001).

## Results

### Decreased m^6^A Level Is Mediated by FTO Upregulation in NPM1-Mutated AML

**Figure 1:**
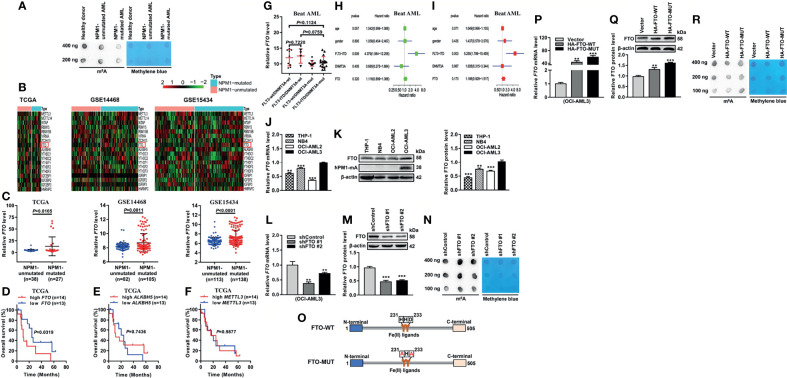
Decreased m^6^A Level is Mediated by FTO Upregulation in NPM1-mutated AML. **(A)** m^6^A dot blot analysis of the global m^6^A abundance in NPM1-mutated AML, NPM1-unmutated AML, and healthy donor samples. Methylene blue staining served as a loading control. **(B)** Heatmaps of m^6^A-related genes in AML patients with NPM1 mutations and without NPM1 mutations from TCGA (n=65), GSE14468 (n=187), and GSE15434 (n=251) datasets. **(C)** The expression patterns of FTO transcript in NPM1-mutated AML samples compared to NPM1-unmutated AML samples from the above three public datasets. **(D–F)** Survival analysis based on *FTO, ALKBH5, METTL3* mRNA expression in TCGA primary NPM1-mutated AML samples. Survival analysis was performed using Kaplan–Meier method. **(G)** The expression patterns of FTO transcript in NPM1-mutated AML samples from Beat AML dataset (n=42). **(H)** Univariate Cox regression analyses of the association between clinicopathological factors (including *FTO* expression) and overall survival of NPM1-mutated AML patients from Beat AML dataset (n=42). **(I)** Multivariate Cox regression analyses of the association between clinicopathological factors (including *FTO* expression) and overall survival of NPM1-mutated AML patients from Beat AML dataset (n=42). **(J, K)** qRT-PCR and western blot analysis of the FTO expression in a panel of human acute myeloid leukemia cell lines. **(L, M)** qRT-PCR and western blot analysis of the FTO mRNA and protein level in OCI-AML3 cells infected with two independent shRNAs targeting FTO or a control shRNA. β-actin was used as the internal control. The bar graph showed the relative level of FTO protein. **(N)** m^6^A dot blot analysis of the global m^6^A abundance in the FTO-silenced OCI-AML3 cells. Methylene blue staining served as a loading control. **(O)** Schematic presentation of the FTO domains and point mutations. **(P, Q)** qRT-PCR and western blot analysis of the FTO mRNA and protein level in OCI-AML3 cells following transfection of the HA-FTO-WT and HA-FTO-MUT plasmids. β-actin was used as the internal control. The bar graph showed the relative level of FTO protein. **(R)** m^6^A dot blot analysis of the global m^6^A abundance in OCI-AML3 cells following transfection of the HA-FTO-WT and HA-FTO-MUT plasmids. Methylene blue staining served as a loading control. Data are presented as the mean ± SD of three independent experiments. **P < 0.01, ***P < 0.001.

To explore whether the abnormal m^6^A level exists in acute myeloid leukemia (AML) with NPM1 mutations, we first detected the m^6^A level of global RNAs. Intriguingly, there was a downregulation of the m^6^A level in primary AML blasts with NPM1 mutations compared with those without NPM1 mutations or the healthy donor sample by m^6^A dot blot assays (**Figure 1A**). Given the pivotal role of m^6^A regulators in m^6^A modifications, we analyzed the expression pattern of m^6^A-related genes by heatmap analysis of the Cancer Genome Atlas (TCGA) and Gene Expression Omnibus (GEO) databases. The results showed that fat mass and obesity-associated protein (FTO) mRNA level was significantly elevated in NPM1-mutated AML cases in comparison with NPM1-unmutated AML cases (**Figures 1B, C**). The Kaplan-Meier analysis revealed that NPM1-mutated AML patients with high *FTO* expression were predicted shorter overall survival, while no prognostic value was shown in other m^6^A catalytic proteins (**Figures 1D–F**). Notably, there was no association between *FTO* expression and molecular features that affect the prognosis of NPM1-mutated AML (FLT3-ITD, and FLT3-ITD and DNMT3A mutations) (**Figure 1G**). Additionally, multivariate Cox regression analyses from Beat AML dataset did not identify FTO as an independent predictor of NPM1-mutated AML (**Figures 1H, I**). Next, the expression of FTO in a panel of AML cell lines was detected by qRT-PCR and western blot analysis. Relatively high expression of FTO mRNA and protein was observed in OCI-AML3 cells naturally carrying NPM1 mutation type A (NPM1-mA) (**Figures 1J, K**). Furthermore, FTO knockdown increased the cellular m^6^A level on the total RNAs (**Figures 1L–N**). In addition, the vectors encoding FTO-WT and FTO-MUT were generated to transfect into OCI-AML3 cells (**Figures 1O–Q**). These results showed downregulation of the global m^6^A abundance in the FTO-WT but not the FTO-MUT group (**Figure 1R**). These results demonstrate that the reduction in the m^6^A level is mediated by FTO upregulation in NPM1-mutated AML.

### High Expression of FTO Is Maintained by NPM1-mA Through Impeding the Proteasome Pathway

**Figure 2:**
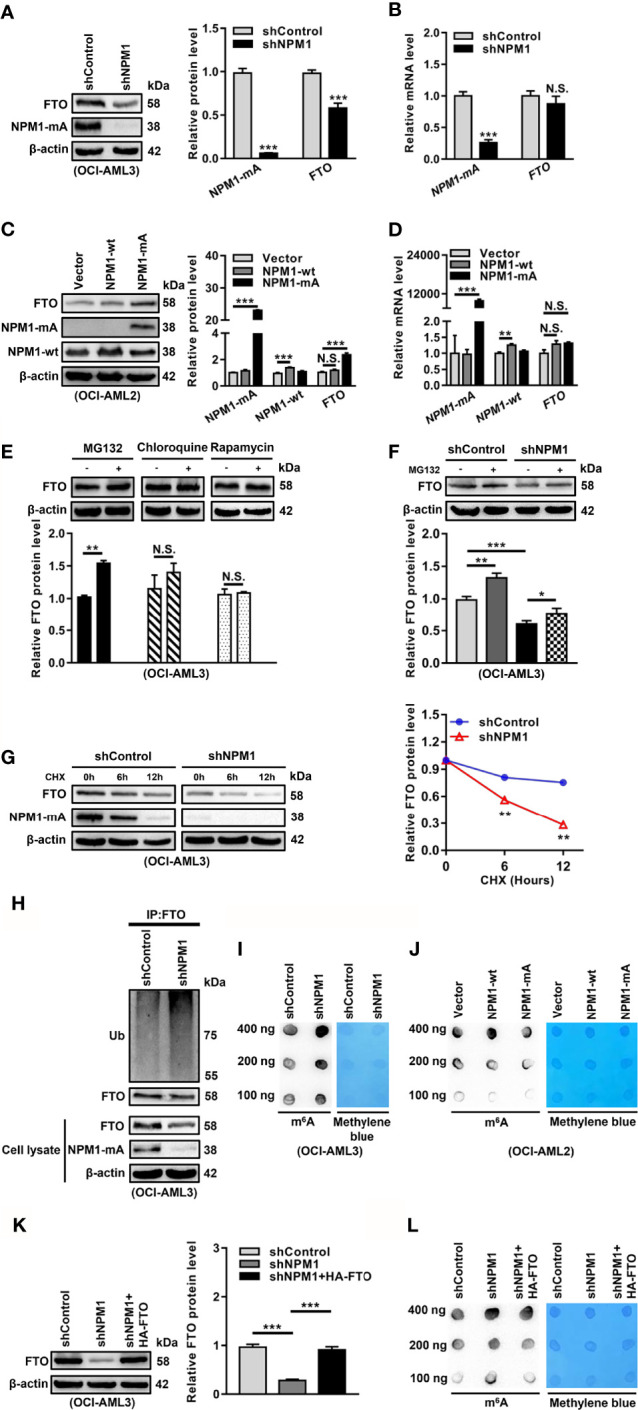
NPM1-mA Upregulates FTO Expression to Reduce the m^6^A Level through Impeding the Proteasome Pathway. **(A, C)** Western blot analysis of the FTO, NPM1-mA, and NPM1-wt protein level in the NPM1-mA-silenced OCI-AML3, NPM1-wt-enforced OCI-AML2, and NPM1-mA-enforced OCI-AML2 cells. The bar graph showed the relative level of protein. **(B, D)** qRT-PCR analysis of the FTO, NPM1-mA, and NPM1-wt mRNA level in the NPM1-mA-silenced OCI-AML3, NPM1-wt-enforced OCI-AML2, and NPM1-mA-enforced OCI-AML2 cells. **(E)** OCI-AML3 cells were treated with the ubiquitin-proteasome pathway inhibitor MG132 (20 μM), the autophagic-lysosomal pathway inhibitor CQ (20 μM), and activator rapamycin (20 μM) for 8 h. The bar graph showed the relative level of FTO protein. **(F)** Western blot analysis of the FTO protein level in the NPM1-silenced OCI-AML3 cells following treatment with MG132 (20 μM) for 8h. The bar graph showed the relative level of FTO protein. **(G)** Western blot analysis of the FTO and NPM1-mA protein level in the NPM1-mA-silenced OCI-AML3 cells following treatment with the protein synthesis inhibitor CHX (100 μg/mL) for the indicated time. The line graph showed the degradation rate of FTO protein. **(H)** Ubiquitination analysis of the ubiquitinated FTO level in the NPM1-mA-silenced OCI-AML3 cells. β-actin was used as the internal control. **(I, J)** m^6^A dot blot analysis of the global m^6^A abundance in the NPM1-mA-silenced OCI-AML3, NPM1-wt-enforced OCI-AML2, and NPM1-mA-enforced OCI-AML2 cells. Methylene blue staining served as a loading control. **(K)** Western blot analysis of the FTO protein level in the NPM1-mA-silenced OCI-AML3 cells transfected with the HA-FTO plasmids. The bar graph showed the relative level of FTO protein. β-actin was used as the internal control. **(L)** m^6^A dot blot analysis of the global m^6^A abundance in the NPM1-mA-silenced OCI-AML3 cells transfected with the HA-FTO plasmids. Methylene blue staining served as a loading control. Data were presented as the mean ± SD of three independent experiments. *P < 0.05, **P < 0.01, ***P < 0.001; N.S. indicated not significant.

Based on the pivotal role of FTO in the abnormal m^6^A level, we aimed to elucidate the reason for FTO upregulation in NPM1-mutated AML. Given that NPM1 mutations were recognized as AML initiating lesions, the regulatory role of NPM1-mA in the FTO expression was investigated. These results showed that the FTO protein level was reduced in the NPM1-mA-silenced OCI-AML3 cells (**Figure 2A**). Conversely, the FTO protein level was increased in the NPM1-mA-enforced OCI-AML2 cells, but no significant change in the NPM1-wt-enforced OCI-AML2 cells (**Figure 2C**). However, the FTO mRNA level remained unchanged upon NPM1-mA knockdown, NPM1-wt overexpression, or NPM1-mA overexpression (**Figures 2B, D**). Thus, whether NPM1-mA was involved in the regulation of FTO protein stability was further explored. Firstly, FTO protein accumulation was observed upon inhibition of the ubiquitin-proteasome pathway by MG132, whereas the autophagic-lysosomal pathway inhibitor chloroquine (CQ) or activator rapamycin had little effect on the FTO protein level (**Figure 2E**). Next, the reduction of the FTO protein level triggered by NPM1-mA knockdown was partially rescued by suppression of the proteasome by MG132 (**Figure 2F**). In addition, treatment with the protein synthesis inhibitor cycloheximide (CHX) revealed that NPM1-mA depletion downregulated the half-life of the FTO protein (**Figure 2G**). Subsequent ubiquitination assays further showed that NPM1-mA knockdown elevated the level of ubiquitinated FTO protein (**Figure 2H**). Of note, NPM1-mA deficiency upregulated the global m^6^A abundance (**Figure 2I**), while NPM1-mA but not NPM1-wt overexpression downregulated the global m^6^A abundance in leukemic cells (**Figure 2J**). More importantly, results from the rescue experiment showed that forced expression of FTO could attenuate NPM1-mA knockdown-induced increase in the global m^6^A abundance (**Figures 2K, L**). These findings demonstrate that high expression of FTO maintained by NPM1-mA reduces the m^6^A level in leukemic cells.

### FTO Promotes Leukemic Cell Proliferation by Facilitating Cell Cycle and Suppressing Cell Apoptosis

**Figure 3:**
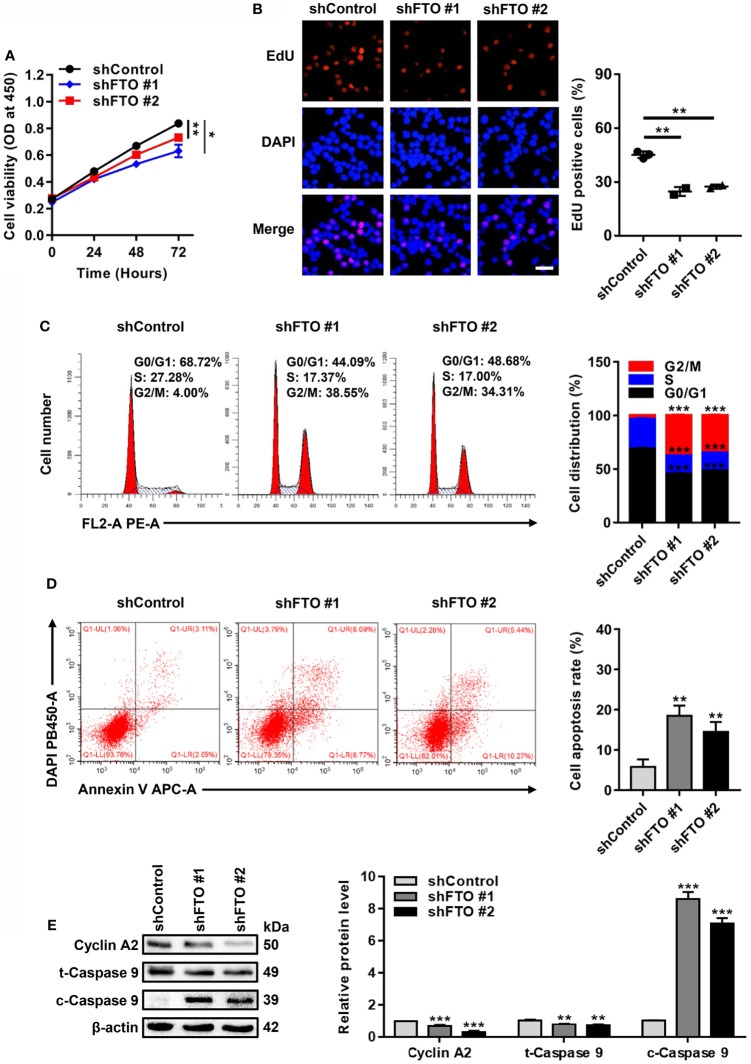
FTO Promotes Leukemic Cell Proliferation by Facilitating Cell Cycle and Suppressing Cell Apoptosis. **(A)** CCK-8 analysis of cell growth in the FTO-silenced OCI-AML3. **(B)** EdU analysis of cell proliferation in the FTO-silenced OCI-AML3 cells. The bar graph showed the percentage of EdU positive cells. Scale bar: 50 μm. **(C)** Flow cytometric analysis of cell cycle distribution in FTO-silenced OCI-AML3 cells. The bar graph showed the percentage of G0/G1, S, and G2/M phase cells. **(D)** Cell apoptosis was measured by flow cytometric analysis of the Annexin V/DAPI stained cells. The bar graph showed the cell apoptosis rate. **(E)** Western blot analysis of Cyclin A2, t-Caspase 9, and c-Caspase 9 protein level in the FTO-silenced OCI-AML3. β-actin was used as the internal control. The bar graph showed the relative level of protein. Data were presented as the mean ± SD of three independent experiments. *P < 0.05, **P < 0.01, ***P < 0.001.

Next, biological functions of FTO upregulation in NPM1-mutated AML were explored. For the loss of function study, CCK-8 assays showed that FTO restraint impaired cell growth (**Figure 3A**). By analogy, a significant reduction in the number of EdU positive cells was visualized in the FTO-silenced OCI-AML3 cells (**Figure 3B**). Flow cytometric analysis of the cell cycle distribution showed a decrease in the fraction of cells in S phase and an increase in that in G2/M phase after FTO knockdown (**Figure 3C**). Meanwhile, FTO deficiency increased the proportion of apoptotic cells (**Figure 3D**). Furthermore, the level of cleaved Caspase 9 (c-Caspase 9) was significantly increased but the level of Cyclin A2 and total Caspase 9 (t-Caspase 9) was decreased in the FTO-silenced OCI-AML3 cells (**Figure 3E**). These results demonstrate that FTO promotes leukemic cell proliferation by facilitating cell cycle and suppressing cell apoptosis.

### The Oncogenic Function of FTO Is Dependent on m^6^A RNA Demethylase Activity

**Figure 4:**
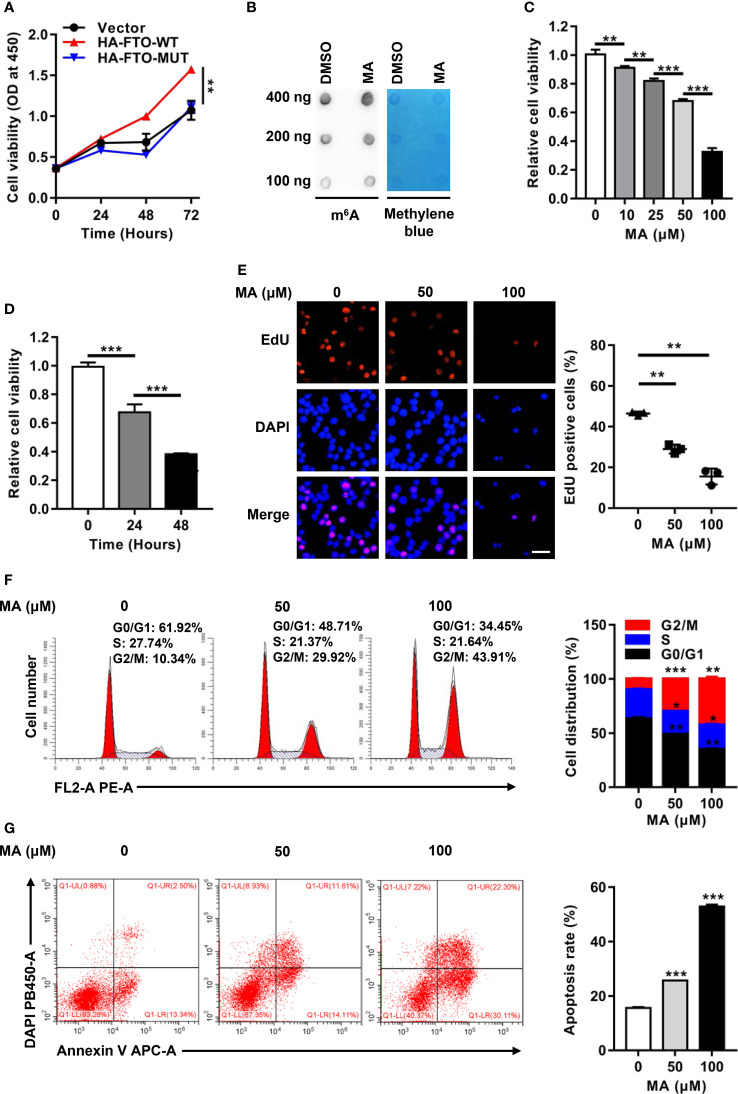
The Oncogenic Function of FTO is Dependent on m^6^A RNA Demethylase Activity. **(A)** CCK-8 analysis of cell proliferation in OCI-AML3 cells following transfection of the HA-FTO-WT and HA-FTO-MUT plasmids. **(B)** m^6^A dot blot analysis of the m^6^A level of global RNAs in OCI-AML3 cells with MA or DMSO treatment for 24 h. Methylene blue staining served as a loading control. **(C)** Dose-dependent effect of MA on cell viability. CCK-8 analysis of cell proliferation in OCI-AML3 cells exposed to 0, 10, 25, 50, and 100 μM MA for 24 h. **(D)** Time-dependent effect of MA on cell viability. CCK-8 analysis of cell proliferation in OCI-AML3 cells exposed to 100 μM MA for 0, 24, and 48 h. **(E)** EdU analysis of cell proliferation in OCI-AML3 cells upon 50 μM and 100 μM MA treatments of 24 h. The bar graph showed the percentage of EdU positive cells. Scale bar: 50 μm. **(F)** Flow cytometric analysis of the cell cycle distribution in OCI-AML3 cells upon 50 μM and 100 μM MA treatments of 24 h. The bar graph showed the percentage of G0/G1, S, and G2/M phase cells. **(G)** Cell apoptosis was measured by flow cytometric analysis of the Annexin V/DAPI stained cells. The bar graph showed the cell apoptosis rate. Data were presented as the mean ± SD of three independent experiments. *P < 0.05, **P < 0.01, ***P < 0.001.

To illustrate how FTO contributed to cell survival, the role of its m^6^A RNA demethylase activity was investigated in NPM1-mutated leukemic cells. Forced expression of FTO-WT, but not FTO-MUT, improved the cell proliferation ability (**Figure 4A**). Next, meclofenamic acid (MA), a selective inhibitor for demethylase activity of FTO, was used to treat OCI-AML3 cells. The results showed that the m^6^A level on the total RNAs was higher in OCI-AML3 cells with MA treatment compared to those with dimethyl sulfoxide (DMSO) treatment (**Figure 4B**). Meanwhile, MA treatments displayed anti-proliferation activity in a dose- and time-dependent manner (**Figures 4C, D**). Correspondingly, MA treatments resulted in a significant decrease in the number of EdU positive cells (**Figure 4E**). In addition, downregulation of the percentage of cells in S phase and upregulation of that in G2/M phase was observed after exposure to 50 μM and 100 μM MA (**Figure 4F**). The cell apoptosis rate was increased following the treatment with MA (**Figure 4G**). These observations suggest that the oncogenic function of FTO is dependent on its m^6^A demethylase activity in NPM1-mutated AML cells.

### FTO Promotes Cell Survival Through PDGFRB/ERK Signaling Axis

**Figure 5:**
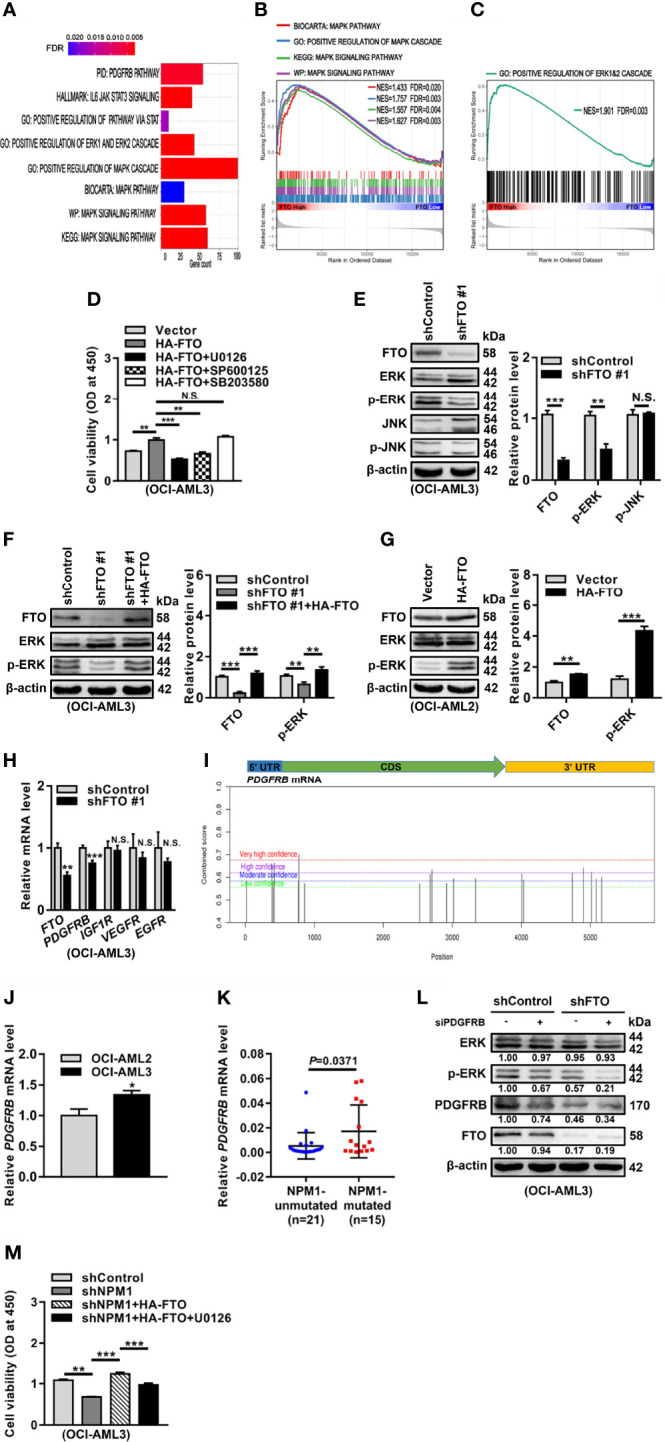
FTO Promotes Cell Survival through PDGFRB/ERK Signaling Axis. **(A–C)** GSEA bar plot showing gene sets enriched in high FTO expression group **(A)**. GSEA enrichment plots of MAPK **(B)** and ERK **(C)** associated gene sets. NES, normalized enrichment score; FDR, false discovery rate. **(D)** OCI-AML3 cells were transfected with the HA-FTO plasmids for 48 h, following treatment with the ERK cascade inhibitor U0126, the JNK cascade inhibitor SP600125, and the p38 cascade inhibitor SB203580 at 5 μM for 24 h, respectively. CCK-8 analysis of cell proliferation in the treated cells. **(E)** Western blot analysis of FTO, ERK, p-ERK, JNK, and p-JNK protein level in the FTO-silenced OCI-AML3 cells. **(F)** The FTO-silenced OCI-AML3 cells were transfected with the HA-FTO plasmids. Western blot analysis of FTO, ERK, and p-ERK protein level in the treated cells. **(G)** Western blot analysis of FTO, ERK, and p-ERK protein level in OCI-AML2 cells transfected with the HA-FTO plasmids. β-actin was used as the internal control. The bar graphs showed the relative level of protein. **(H)** qRT-PCR analysis of the FTO, PDGFRB, IGF1R, VEGFR, and EGFR mRNA level in the FTO-silenced OCI-AML3 cells. **(I)** Prediction score of m^6^A distribution in PDGFRB mRNA sequence using SRAMP. **(J)** qRT-PCR analysis of the PDGFRB mRNA level in OCI-AML2 and OCI-AML3 cells. **(K)** qRT-PCR analysis of the PDGFRB mRNA level in primary AML blasts without (n=21) and with (n=15) NPM1 mutations. **(L)** Western blot analysis of the FTO, ERK, p-ERK, and PDGFRB protein level in the FTO-silenced OCI-AML3 cells after PDGFRB knockdown. β-actin was used as the internal control. **(M)** CCK-8 analysis of cell proliferation in the NPM1-mA-silenced OCI-AML3 cells transfected with the HA-FTO plasmids for 48 h, following the treatment with 5 μM U0126 for 24 h. Data were presented as the mean ± SD of three independent experiments. *P < 0.05, **P < 0.01, ***P < 0.001; N.S. indicated not significant.

To further dissect the molecular mechanisms underlying the oncogenic role of FTO, we first explored the potential signaling pathways related to FTO expression in NPM1-mutated AML by Gene Set Enrichment Analysis (GSEA). These data showed that the mitogen-activated protein kinase (MAPK) signaling pathway and extracellular signal-regulated kinase (ERK)1/2 cascade were enriched in high FTO expression group (**Figures 5A–C**). It is well known that ERK cascade belongs to MAPK signaling pathways, which also include c-Jun N-terminal kinase (JNK) and p38 cascades (38). In the study, treatment with the ERK cascade inhibitor (U0126) or the JNK cascade inhibitor (SP600125) reduced the effect of FTO overexpression on cell viability, while no significant decrease in the group treated with the p38 cascade inhibitor (SB203580) (**Figure 5D**). However, the level of phosphorylated ERK (p-ERK) but not phosphorylated JNK (p-JNK) was downregulated in the FTO-silenced OCI-AML3 cells (**Figure 5E**). Furthermore, the decreased p-ERK level triggered by FTO depletion was restored after forced expression of FTO (**Figure 5F**). Conversely, introduction of HA-FTO plasmids into OCI-AML2 cells upregulated the level of p-ERK (**Figure 5G**). Next, the expression of the upstream molecules of ERK cascade was detected upon FTO knockdown. These results revealed that the mRNA level of platelet-derived growth factor receptor beta (PDGFRB), but not the insulin-like growth factor 1 receptor (IGF1R), vascular endothelial growth factor receptor (VEGFR), and epidermal growth factor receptor (EGFR) mRNA level, was decreased after FTO depletion (**Figure 5H**). Additionally, several m^6^A methylated sites were identified in the *PDGFRB* mRNA sequence using the sequence-based RNA adenosine methylation site predictor (SRAMP) database (**Figure 5I**). Relatively high expression of PDGFRB mRNA was subsequently confirmed by qRT-PCR in NPM1-mA-positive OCI-AML3 cells and primary AML blasts (**Figures 5J, K**). More importantly, the results showed that PDGFRB knockdown decreased the p-ERK level in FTO-silenced OCI-AML3 cells (**Figure 5L**). Then, the role of FTO-mediated activation of ERK cascade in NPM1-mA oncogenic function was investigated. The results revealed that the ERK cascade inhibitor (U0126) hindered the effect of FTO on NPM1-mA-mediated growth advantage (**Figure 5M**). These data showed that FTO promotes cell survival through PDGFRB/ERK signaling axis, which is required for NPM1-mA pro-survival role.

## Discussion

**Figure 6:**
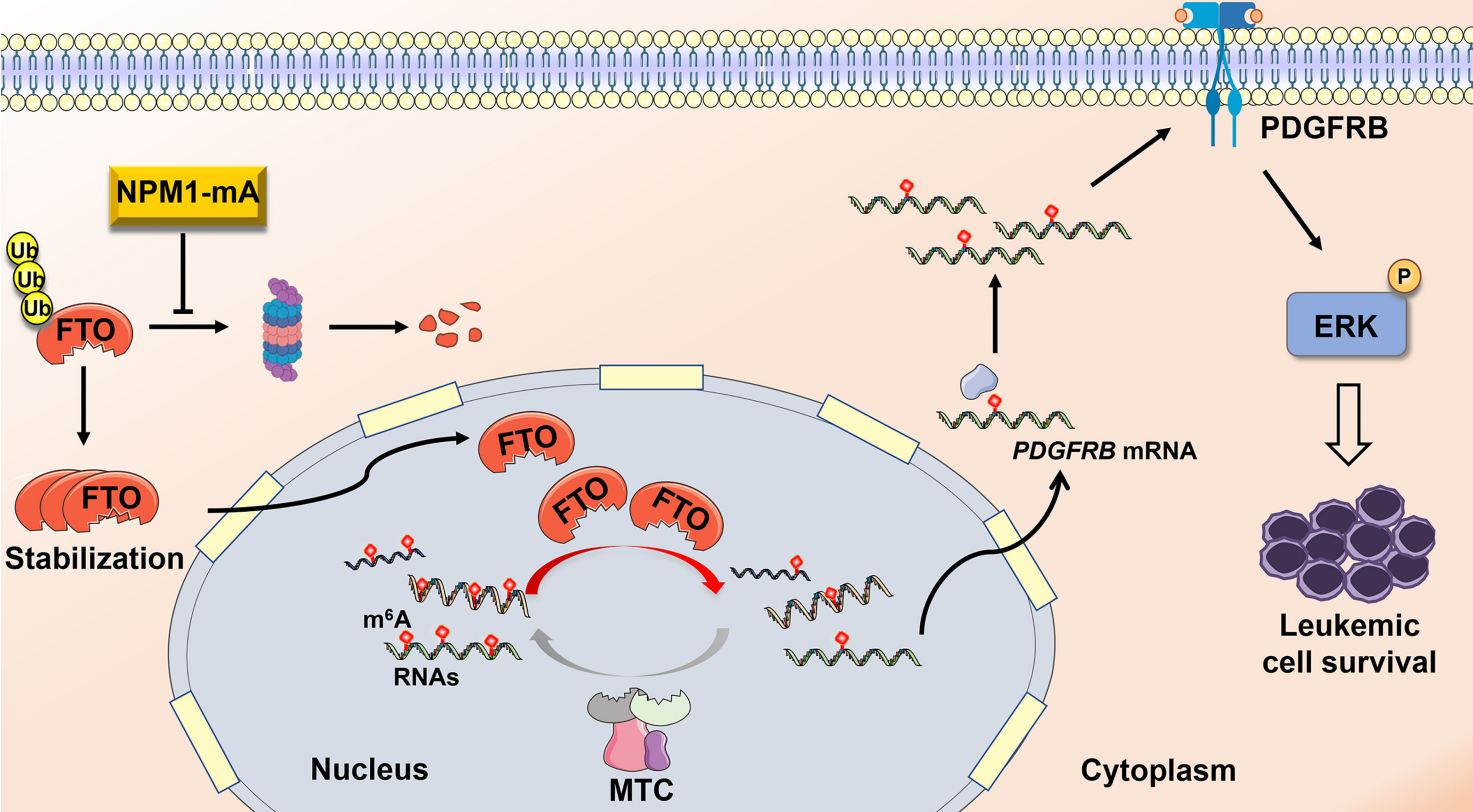
Schematic diagram illustrating the functional significance of FTO-mediated m^6^A demethylation in NPM1-mutated leukemic cells. NPM1-mA stabilizes the FTO protein to reduce the global m^6^A abundance, leading to activation of PDGFRB/ERK signaling axis and further promoting leukemic cell survival.

Compelling evidence has corroborated that disruption of m^6^A methylation distribution in RNAs is a hallmark of various cancers, leading to tumorigenesis and development (39, 40). However, the role of *N*^6^-methyladenosine (m^6^A) modifications in NPM1-mutated AML remains poorly understood. Herein, our data demonstrate that fat mass and obesity-associated protein (FTO) upregulation induced by NPM1-mA accounts for the decreased m^6^A level in leukemic cells. Furthermore, FTO activated PDGFRB/ERK signaling axis by its m^6^A RNA demethylase activity, thereby contributing to leukemic cell survival (**Figure 6**).

In the present study, the reduced global m^6^A level was first detected in the NPM1-mutated AML case compared with the healthy donor or the NPM1-unmutated AML case. Next, we attempted to identify differentially expressed m^6^A regulators in NPM1-mutated AML. Relatively high level of the m^6^A demethylase FTO transcript and protein was observed in AML with NPM1 mutations by the analysis of three open datasets and a panel of AML cell lines. Notably, a subset of NPM1-mutated AML patients from TCGA and GEO databases did not appear high FTO expression compared with NPM1-unmutated AML patients, which might be as a result of the regulation of FTO expression by multiple factors such as estrogen, total fatty acid intake in diet, and arsenic exposure, and so on and the highly heterogeneity of AML (41–43). Indeed, Li et al. (44) also found that FTO was expressed at a significantly higher level in NPM1-mutated AML. In our study, knockdown of FTO upregulated the m^6^A level, whereas overexpression of FTO had the opposite effect. These data suggest that the reduced global m^6^A abundance in NPM1-mutated AML is at least partially associated with the upregulation of FTO expression. In fact, the deposition of m^6^A modifications is highly a dynamic and reversible progress regulated by a variety of methyltransferases and demethylases (45). Recently, a study revealed that the methyltransferase METTL14 downregulation was responsible for the decreased m^6^A modification in bladder tumor-initiating cells (46). Guo et al. demonstrated that low expression of the demethylase ALKBH5 led to upregulation of the m^6^A level in pancreatic cancer (47). Thus, further study deserves performing to explore the significances of other m^6^A regulators for the aberrant m^6^A abundance in NPM1-mutated AML.

Given the essential role of NPM1-mA in leukemogenesis and development, we investigated whether this mutation regulated the expression of FTO to affect the m^6^A level. In this work, knockdown of NPM1-mA decreased and overexpression of NPM1-mA increased the FTO protein level, whereas NPM1-mA did not change the FTO mRNA level. The existing study reported that FTO was degraded by selective autophagy in keratinocytes (43). The proteasome pathway was involved in the FTO protein stability in HeLa cells (48). In our study, NPM1-mA stabilized the FTO protein by inhibiting the ubiquitin-proteasome pathway. Our previous study showed that NPM1-mA maintained the Kruppel like factor 5 (KLF5) protein stability through diminishing the E3 ubiquitin ligase WW Domain-Containing Protein 1 (WWP1)-induced ubiquitination (49). Notably, Ruan et al. (50) reported that the reduction in the FTO protein level was mainly due to the overexpression of the E3 ubiquitin ligase serine/threonine kinase receptor-associated protein (STRAP) in colorectal cancer cells. Additionally, the FTO protein level was positively correlated with the expression of the deubiquitinase ubiquitin specific peptidase 18 (USP18) expression in bladder cancer (51). Therefore, further work is needed to investigate which ubiquitin-related regulators are responsible for the effect of NPM1-mA on the FTO proteasomal degradation process. Interestingly, the results from our experiments revealed that NPM1-mA reduces the global m^6^A abundance by upregulating FTO expression, suggesting the possible link between NPM1-mA and the aberrant m^6^A modification in leukemia. In fact, the cellular m^6^A level is susceptible to diverse factors. Zhang et al. (52) reported that under hypoxic conditions, HIF-dependent increase of ALKBH5 expression promoted the m^6^A demethylation in breast cancer cells. In another study, upregulation of the m^6^A level in mRNAs was observed after exposure of human bronchial epithelial cells to cigarette smoke extract (53). Thus, a deeper exploration of other regulatory mechanisms contributing to the global m^6^A abundance in NPM1-mutated AML is imperative.

FTO, as the first identified m^6^A RNA demethylase (20), has been widely known to be implicated in cancer progression and clinical outcomes (54–56). In this work, we verified that FTO promoted leukemic cell proliferation by facilitating cell cycle and inhibiting cell apoptosis. Of note, Li et al. (44) revealed that FTO improved the colony-forming potential of mouse leukemic cells carrying FLT3-ITD and NPM1 mutant. FTO also served as an oncogene in glioblastoma progression (57). In addition, depletion of FTO led to the compromised proliferation of pancreatic cancer cells (58). It has been well known that the m^6^A RNA demethylase activity of FTO is involved in its oncogenic role (59, 60). In our study, forced expression of FTO-WT, but not FTO-MUT, accelerated the leukemic cell growth. Furthermore, treatments with meclofenamic acid (MA), a highly selective inhibitor for FTO (61), increased the m^6^A level of global RNAs, thereby restraining cell survival. Notably, a study showed that inhibition of FTO by CS1/CS2 dramatically attenuated leukemia stem/initiating cell self-renewal and reprogrammed immune response (62). Our observations and these previous reports indicate that targeting the m^6^A demethylation mediated by FTO might offer a promising avenue to treat NPM1-mutated AML. Recent advances have illuminated that the m^6^A regulators maintain the cancer-related biological processes through the regulation of different signal transduction pathways (63, 64). In our study, we first focused on MAPK/ERK-associated gene sets by bioinformatics analysis. Then, the ERK cascade inhibitor (U0126) could abolish the proliferative effect of FTO on leukemic cells, and the level of p-ERK was positively regulated by FTO. Given that receptor tyrosine kinases (RTKs) play important roles in the initiation of ERK cascade (65), whether RTKs contribute to FTO-mediated activation of ERK cascade was explored. PDGFRB, but not the other relevant membrane RTKs mRNA level, was downregulated after FTO depletion. In addition, m^6^A methylated sites were identified in the PDGFRB mRNA sequence according to the SRAMP database. High expression of PDGFRB mRNA was subsequently confirmed by qRT-PCR in NPM1-mA-positive OCI-AML3 cells and primary AML blasts. More importantly, PDGFRB knockdown decreased the p-ERK level in FTO-silenced OCI-AML3 cells. These data indicate that FTO accelerates cell proliferation through activating PDGFRB/ERK signaling axis in NPM1-mutated AML. It has been reported that another PDGFRB downstream signaling, PI3K-AKT cascade, participates in leukemic cell proliferation advantage (66, 67). Moreover, a recent study has revealed that FTO overexpression could activate PI3K-AKT cascade in bone marrow mesenchymal stem cells (68). Therefore, whether PI3K-AKT signaling is involved in FTO-mediated leukemic cell proliferation advantage is necessary to be further investigated. Additionally, a recent study demonstrated that FTO evoked Wnt/β-catenin pathway by stabilizing the frizzled class receptor 10 (FZD10) mRNA in BRCA-mutated epithelial ovarian cancer (69). FTO inactivated p53 pathway to accelerate the proliferation rate of lung cancer cells through the upregulation of USP7 transcript (70). Thus, a deeper exploration of other signaling pathways involved in the biological functions of FTO in NPM1-mutated AML is imperative. Finally, we verified that FTO-mediated activation of ERK cascade was crucial to NPM1-mA pro-survival function. In our previous study, NPM1-mA enhanced the ERK phosphorylation level through the interaction with K-Ras (71). Collectively, our findings provide a new insight that FTO-mediated m^6^A demethylation is expected to be a novel therapeutic target for NPM1-mutated AML. In fact, further work is needed to elucidate the role of FTO-mediated activation of ERK cascade in mouse knock-in models that mimic human NPM1-mutated AML.

## Data Availability Statement

The datasets presented in this study can be found in online repositories. The names of the repository/repositories and accession number(s) can be found in the article/supplementary material.

## Ethics Statement

The studies involving human participants were reviewed and approved by the Ethics Committee of Chongqing Medical University and Chongqing University. The patients/participants provided their written informed consent to participate in this study.

## Author Contributions

LZ and QX initiated the work and designed the experiments. QX performed the experiments and wrote the manuscript. MP, YJ, and XJ contributed techniques and commented on the manuscript. YT and JR analyzed the data. LL and JH contributed analytic tools. ZY provided clinical assistance. CL, JY, MS, LT, and XW assisted with revising the manuscript. All authors read and approved the final manuscript.

## Funding

This work was supported by the National Natural Science Foundation of China (NSFC81873973 and NSFC82072353) and the Natural Science Foundation of CQ CSTC (cstc2019jcyj-msxmX0229 and cstc2021jcyj-msxmX0363).

## Conflict of Interest

The authors declare that the research was conducted in the absence of any commercial or financial relationships that could be construed as a potential conflict of interest.

## Publisher’s Note

All claims expressed in this article are solely those of the authors and do not necessarily represent those of their affiliated organizations, or those of the publisher, the editors and the reviewers. Any product that may be evaluated in this article, or claim that may be made by its manufacturer, is not guaranteed or endorsed by the publisher.

## Glossary

AML: acute myeloid leukemia
NPM1: nucleophosmin 1
NPM1-mA: NPM1 mutation type A
m^6^A: *N*^6^-methyladenosine
FTO: fat mass and obesity-associated protein
ALKBH5: alkB homolog 5
METTL3: methyltransferase-like 3
METTL14: methyltransferase-like 14
MAPK: mitogen-activated protein kinase
ERK: extracellular signal-regulated kinase
JNK: c-Jun N-terminal kinase
GEO: Gene Expression Omnibus
TCGA: the Cancer Genome Atlas
DSMZ: Deutsche Sammlung von Mikroorganismen und Zellkulturen GmbH
ATCC: American Type Culture Collection
WHO: World Health Organization
FBS: fetal bovine serum
qRT-PCR: quantitative real-time PCR
shRNA: short hairpin RNA
siRNA: short interfering RNA
SDS-PAGE: sodium dodecyl sulfate-polyacrylamide gel electrophoresis
PVDF: polyvinylidene fluoride
DMSO: dimethyl sulfoxide
CHX: cycloheximide
CQ: chloroquine
MA: meclofenamic acid
CCK-8: Cell Counting Kit-8
EdU: 5-ethynyl-2’-deoxyuridine
DAPI: 4’,6-diamidino-2-phenylindole
SD: standard deviation
PBS: phosphate-buffered saline
TBS: tris-buffered saline
RTK: receptor tyrosine kinase
PDGFRB: platelet-derived growth factor receptor beta
IGF1R: insulin-like growth factor 1 receptor
VEGFR: vascular endothelial growth factor receptor
EGFR: epidermal growth factor receptor
GSEA: Gene Set Enrichment Analysis
MSigDB: Molecular Signatures Database
GMT: Gene Matrix Transposed
NES: normalized enrichment score
FDR: false discovery rate
SRAMP: sequence-based RNA adenosine methylation site predictor
PI: propidium
